# Potassium Measures and Their Associations with Glucose and Diabetes Risk: The Multi-Ethnic Study of Atherosclerosis (MESA)

**DOI:** 10.1371/journal.pone.0157252

**Published:** 2016-06-09

**Authors:** Ranee Chatterjee, Leila Zelnick, Kenneth J. Mukamal, Jennifer A. Nettleton, Bryan R. Kestenbaum, David S. Siscovick, Joachim H. Ix, Russell Tracy, Andrew N. Hoofnagle, Laura P. Svetkey, David Edelman, Ian H. de Boer

**Affiliations:** 1 Department of Medicine, Duke University, Durham, NC, United States of America; 2 Department of Biostatistics, University of Washington, Seattle, WA, United States of America; 3 Department of Medicine, Beth Israel Deaconess Medical Center, Harvard Medical School, Boston, MA, United States of America; 4 Division of Epidemiology, The University of Texas Health Sciences Center, Houston, TX, United States of America; 5 Department of Medicine, University of Washington, Seattle, WA, United States of America; 6 The New York Academy of Medicine, New York, NY, United States of America; 7 Department of Medicine, University of California San Diego and Veterans Affairs San Diego Healthcare System, San Diego, CA, United States of America; 8 Department of Pathology, University of Vermont, Burlington, VT, United States of America; 9 Department of Lab Medicine, University of Washington, Seattle, WA, United States of America; 10 Department of Epidemiology, University of Washington, Seattle, WA, United States of America; University Heart Center, GERMANY

## Abstract

**Background:**

Recent studies have found low-normal potassium (K) to be associated with increased diabetes risk. We sought to verify these associations in a multi-ethnic US cohort; and to determine if these associations extend to US Hispanics and Asian-Americans.

**Methods:**

We analyzed data from Multi-Ethnic Study of Atherosclerosis (MESA) participants who were free-of-diabetes at baseline. We examined cross-sectional associations between measures of K—serum, dietary, and urine—with fasting glucose and HOMA-IR. We examined longitudinal associations between K and diabetes risk over 8 years.

**Findings:**

In multivariable models, compared to those with higher serum K (≥4.5mmol/L), those with lower serum K (<4.0mmol/L) had significantly higher fasting glucose [1.3 mg/dL (95%CI 0.2, 2.4), *P*-value = 0.03]. Incident diabetes developed in 1281 of 5415 at-risk participants. In minimally-adjusted models, we found inverse associations between serum and dietary K and diabetes risk. Compared to those with higher serum K, those with lower serum K had an HR (95% CI) of incident diabetes of 1.23 (1.04, 1.47), *P*-value = 0.02. However, these associations were attenuated in fully-adjusted models. We found no significant interaction between potassium and ethnicity.

**Conclusions:**

In this multi-ethnic cohort, we found a significant inverse association between serum K and fasting glucose but no significant association with longer-term diabetes risk. This inverse association between potassium and glucose must be studied further to understand the physiology and its potential impact on chronic health.

## Introduction

In secondary data analyses of certain US and international cohorts, low serum and low dietary potassium have been associated with higher risk of diabetes.[1–3] Adults in the US generally consume less than the recommended amount of dietary potassium, with one study finding that less than 2% of adults consumed the recommended amount of ≥4700mg/day.[4] There are also known disparities in dietary potassium intake between African Americans and whites, with African Americans consuming lower amounts of dietary potassium compared to whites.[4] In some studies, both serum and dietary potassium (K) have shown stronger associations with diabetes risk in African Americans compared to whites.[2, 5] Low serum K has been associated with increased diabetes risk and increased prevalence of metabolic syndrome in some Asian populations.[3, 6] Experimental studies provide biological plausibility to the association between low K and abnormal glucose metabolism, by showing that thiazide-induced hypokalemia leads to diminished insulin secretion.[7, 8] Genetic mutations in K channels on pancreatic β-cells are associated with increased diabetes risk, also suggesting a potential biological basis for the association between K and glucose metabolism.

While there has been some exploration of differences in potassium and health effects in African Americans and whites, these explorations have not extended to other ethnicities in the US. Using data from the Multi-ethnic Study of Atherosclerosis (MESA) cohort, a cohort which includes Hispanics and Chinese Americans, we sought to determine the associations between potassium measures with measures of glucose metabolism and diabetes risk.

## Methods

### Study population

MESA is a prospective community-based cohort of adults aged 45–84 years of age at baseline. Baseline data were collected between July 2000 and August 2002 with 4 subsequent in-person exams occurring through 2012. Participants were recruited from 6 different communities: Baltimore City and County, MD; Chicago, IL, Forsyth County, NC, Los Angeles County, CA, Northern Manhattan and Bronx, NY. Four ethnicities were represented in this cohort: of the 6814 participants recruited at baseline, 38% were white, 28% were African American, 22% Hispanic, and 12% Asian, predominantly of Chinese descent. Institutional review boards (IRB) at each of the participating institutions for MESA approved the study, and written informed consent was obtained from each participant. Additionally, the IRBs at Duke University and the University of Washington approved the conduct of the data analyses presented in this manuscript.

Participants were excluded from these analyses if they had diabetes mellitus evident at the baseline exam (n = 859). Diabetes was defined as fasting serum glucose ≥126mg/dL and/or use of glucose-lowering medications. We additionally excluded any remaining participants who were missing any of the K exposures (n = 540).

### Exposures

Serum K was measured utilizing indirect potentiometry with an ion-selective electrode. Dietary K intake was derived from a modified Block-style 120-item food frequency questionnaire (FFQ) administered at the baseline exam. This FFQ was initially designed for the Insulin Resistance and Atherosclerosis Study (IRAS) cohort study but was modified to accommodate the diets of the different ethnicities included in the MESA cohort. From this FFQ, individual nutrient intake was quantified using the Nutrition Data System for Research (NDS-R database; Nutrition Coordinating Center, Minneapolis, MN, USA).[9] The coordinating center updated the nutrient variables in June 2015 in order to correct a coding error. This error was found in a subset of participants, depending on participant response to a modifier question, to lead to at least some underreporting of certain food frequency, food group, and nutrient variables. Original data was used to update some of these errors when available, and single imputation methods were used to correct the remaining errors when original data was not available.

Urinary K was measured from a random spot specimen on a Beckman Coulter chemistry analyzer (DXC 600) using indirect potentiometry utilizing a potassium ion- selective electrode in conjunction with a sodium reference electrode. Fractional excretion of K (FEK) was calculated as (urine K/urine Cr)/(serum K/serum Cr). This was presumed to reflect the renal handling of filtered K, as influenced by aldosterone and the delivery of sodium and K to the distal renal tubules.

All exposures were categorized for analyses. Serum K was categorized by clinically relevant cut-points or ≥4.5, 4.0–4.49, and <4.0mmol/L. Dietary K, urinary K, and FEK were categorized into quartiles.

### Outcomes

For cross-sectional analyses, outcomes were fasting serum glucose and HOMA-IR, a measure of insulin resistance which was calculated as (fasting glucose*fasting insulin)/405, both measured at the baseline exam. Serum glucose was measured using the glucose oxidase method on the Vitros analyzer (Johnson & Johnson Clinical Diagnostics, Inc, Rochester, NY). Insulin was measured by a radioimmunoassay method using the Linco Human Insulin Specific RIA Kit (Linco Research, Inc., St. Charles, MO).

For longitudinal analyses, incident diabetes mellitus was defined as fasting serum glucose ≥126mg/dL and/or use of glucose-lowering medications at any of the follow-up exams through 2012 for a mean of 8 years of follow-up. Baseline exam was performed between the years 2000–2002; there were 3 additional follow-up exams between baseline exam and the year 2007, approximately 18–24 months apart; and a fifth in-person visit between 2010–2012.

### Covariates

Covariate data were collected at the baseline exam through a combination of self-administered and interviewer-administered questionnaires as well as in-person measurements. Waist circumference was measured at the umbilicus using a measuring tape and rounding to the nearest centimeter. Body-mass index was calculated using height and weight measurements from a stadiometer and calibrated scale. Physical activity was measured using the MESA Typical Week Physical Activity Survey, which was adapted from the Cross-Cultural Activity Participation Survey.[10] From this survey, physical activity was quantified and categorized as minutes spent on activities of different metabolic equivalents. Systolic blood pressure was calculated as the average of the second and third blood pressure readings with patient in a seated position and with readings taken 5 minutes apart.

### Statistical methods

Baseline characteristics were evaluated by categories of each exposure of interest. Correlations between exposures were assessed. Multivariable linear regression models were used to test cross-sectional associations with the outcomes of fasting glucose or HOMA-IR. Multivariable discrete Cox regression models were used to test associations with incident diabetes. We included covariates which were thought to potentially affect relationships between K and glucose metabolism based on current understanding of pathophysiology. Model 1 adjusted for age (continuous), sex, race/ethnicity, study site, total energy intake (for dietary analyses only), and log of urinary creatinine (for urine K analyses only). Model 2 additionally adjusted for waist circumference (continuous), body mass index (continuous), smoking, family history of diabetes, education (categories), income (categories), alcohol use (categories), and physical activity (categories), systolic blood pressure, and use of antihypertensive medications (as listed in Table 1). We built parallel models with and without interaction terms for race/ethnicity x K exposure in order to evaluate associations for the full population and for participants of each race/ethnicity, respectively. We also tested for statistical interactions in the full cohort between hypertension status/use of anti-hypertensive medications and K exposure. Wald tests were used to test for heterogeneity. Tests of significance were two-tailed with an α level of 0.05. We performed all analyses using R version 3.2.0.[11]

**Table 1:** Baseline Characteristics of Study Population (N = 5415), by Serum K (mmol/L).

Covariate	All (N = 5415)	K < 4.0 (N = 804)	4.0 ≤ K < 4.5 (N = 2844)	K ≥ 4.5 (N = 1767)
**Demographics**				
Age (years)	61.8 ± 10.3	62.1 ± 10.0	61.5 ± 10.2	62.3 ± 10.5
Sex				
Female	2924 (54.0)	514 (63.9)	1574 (55.3)	836 (47.3)
Male	2491 (46.0)	290 (36.1)	1270 (44.7)	931 (52.7)
Race/Ethnicity				
White, Caucasian	2281 (42.1)	238 (29.6)	1117 (39.3)	926 (52.4)
Chinese-American	679 (12.5)	160 (19.9)	435 (15.3)	84 (4.8)
Black, African-American	1345 (24.8)	270 (33.6)	707 (24.9)	368 (20.8)
Hispanic	1110 (20.5)	136 (16.9)	585 (20.6)	389 (22.0)
Site				
Wake Forest	828 (15.3)	131 (16.3)	395 (13.9)	302 (17.1)
Columbia	827 (15.3)	85 (10.6)	392 (13.8)	350 (19.8)
Johns Hopkins	807 (14.9)	104 (12.9)	400 (14.1)	303 (17.1)
Minnesota	869 (16.0)	91 (11.3)	437 (15.4)	341 (19.3)
Northwestern	1044 (19.3)	219 (27.2)	651 (22.9)	174 (9.8)
UCLA	1040 (19.2)	174 (21.6)	569 (20.0)	297 (16.8)
**Anthropometric measures:**				
Body mass index (kg/m^2^)	27.9 ± 5.3	28.0 ± 5.7	27.8 ± 5.3	28.0 ± 5.0
Waist circumference (cm)	96.8 ± 14.0	96.6 ± 14.9	96.3 ± 13.8	97.7 ± 13.7
**Lifestyle and family history:**				
Smoking status				
Never	2762 (51.0)	450 (56.0)	1509 (53.1)	803 (45.4)
Former	1967 (36.3)	265 (33.0)	1004 (35.3)	698 (39.5)
Current	679 (12.5)	87 (10.8)	328 (11.5)	264 (14.9)
Family history of DM	2086 (38.5)	316 (39.3)	1096 (38.5)	674 (38.1)
Education				
Less than high school degree	881 (16.3)	135 (16.8)	448 (15.8)	298 (16.9)
High school to some college	1821 (33.6)	265 (33.0)	941 (33.1)	615 (34.8)
College degree or higher	2705 (50.0)	402 (50.0)	1451 (51.0)	852 (48.2)
Total gross family income, $				
< 20,000	1850 (34.2)	272 (33.8)	995 (35.0)	583 (33.0)
20,000–49,999	2099 (38.8)	340 (42.3)	1064 (37.4)	695 (39.3)
≥ 50,000	1290 (23.8)	160 (19.9)	691 (24.3)	439 (24.8)
Number of alcoholic drinks/week				
0	1313 (24.2)	185 (23.0)	706 (24.8)	422 (23.9)
1–7	1539 (28.4)	213 (26.5)	801 (28.2)	525 (29.7)
≥ 7	549 (10.1)	45 (5.6)	284 (10.0)	220 (12.5)
Physical activity				
High activity	1312 (24.2)	183 (22.8)	650 (22.9)	479 (27.1)
Moderate activity	2796 (51.6)	405 (50.4)	1496 (52.6)	895 (50.7)
Light activity	1302 (24.0)	214 (26.6)	696 (24.5)	392 (22.2)
**Medical history:**				
Systolic blood pressure (mmHg)	125.4 ± 21.2	132.1 ± 23.4	124.4 ± 20.6	124.0 ± 20.5
Diastolic blood pressure (mmHg)	71.8 ± 10.2	74.1 ± 11.1	71.7 ± 10.1	70.8 ± 9.9
Fasting serum glucose (mg/dL)	89.5 ± 10.5	90.7 ± 11.4	89.4 ± 10.5	89.2 ± 10.2
Antihypertensive medication use	1789 (33)	431 (54)	850 (30)	508 (29)
ARB or ACE-I use	749 (13.8)	157 (19.5)	337 (11.8)	255 (14.4)
Thiazide diuretics w/o K-sparing agents	351 (6.5)	164 (20.4)	132 (4.6)	55 (3.1)
Thiazide diuretics w/ K-sparing agents	203 (3.7)	80 (10.0)	84 (3.0)	39 (2.2)
Loop diuretics	79 (1.5)	18 (2.2)	31 (1.1)	30 (1.7)
Potassium supplements	97 (1.8)	50 (6.2)	36 (1.3)	11 (0.6)
Potassium-sparing agents	28 (0.5)	7 (0.9)	12 (0.4)	9 (0.5)
eGFR CKD-EPI (mL/min/1.73m^2^)	77.8 ± 15.5	79.0 ± 16.2	79.1 ± 14.9	75.3 ± 15.9
Dietary potassium intake (mg/day)	2847 ± 1458	2676 ±1407	2827 ±1466	2956 ±1459

Entries are mean ± standard deviation or number (%)

ARB- angiotensin II receptor antagonists; ACE-I- angiotension converting enzyme-inhibitors

GFR = glomerular filtration rate

## Results

### Baseline characteristics

Table 1 shows baseline characteristics of the 5415 included participants by serum K categories. S4 Appendix Table shows baseline characteristics of the participants who were excluded from our analyses. Of the four ethnicities represented in this cohort, whites had the greatest proportion with a high-normal serum K (≥4.5mmol/L) (52%), while African Americans had the greatest proportions with a low-normal serum K (<4.0mmol/L) (34%). Compared to those with a high-normal serum K, participants with a low-normal serum K had a higher systolic blood pressure and greater use of the blood pressure medications that affect renal K handling. Participants in all 3 categories of serum K had similar mean BMI. Correlations between exposures were found to be fairly low. The correlation between serum K and dietary K was 0.04; the correlation between serum K and urinary K was 0.15; and the correlation between dietary K and urinary K was 0.03.

### Fasting glucose and HOMA-IR

In cross-sectional analyses using the baseline data, we did find a significant inverse association between serum K and fasting glucose.(Table 2) In our fully-adjusted model (model 2), compared to those with a high-normal serum K (≥4.5mmol/L), those with a low-normal serum K (<4.0mmol/L) had a higher fasting glucose (95% CI) of 1.3mg/dL (0.2, 2.4), *P*-value = 0.03. There was no statistical interaction between serum K and ethnic group on this outcome (*P* = 0.53) There was also no statistical interaction between serum K and hypertension status on this outcome (*P* = 0.46). The direction of the association was consistent in all ethnic groups, except for the Chinese Americans.(Table 2) In minimally-adjusted models (model 1), we found significant inverse associations between dietary K and urinary FEK with fasting glucose; however, after further adjustment, these associations were no longer significant. (S1 and S2 Appendix Tables) We found no significant associations between random urinary K and fasting glucose.

**Table 2:** Associations Between Serum Potassium and Fasting Glucose, by Ethnicity (N = 5415).

			Adjusted difference (95% CI) (mg/dL)	
Serum K (mmol/L)	N	Mean glucose (mg/dL)	Model 1	Model 2
**All participants**	5415	89.5 (10.5)		
< 4.0	1767	90.7 (11.4)	**1.7 (0.8, 2.6)**	**1.3 (0.2, 2.4)**
4.0–4.49	804	89.4 (10.5)	0.4 (-0.2, 1.0)	0.3 (-0.5, 1.0)
≥ 4.5	2844	89.2 (10.2)	0 (Ref.)	0 (Ref.)
per 0.5 lower			**1.1 (0.7, 1.5)**	**0.6 (0.1, 1.0)**
p-value			**< 0.0001**	**0.03**
**White**	2281	87.8 (10.1)		
< 4.0	238	89.2 (11.4)	**2.4 (1.0, 3.9)**	**1.8 (0.3, 3.3)**
4.0–4.49	1117	87.6 (10.2)	0.4 (-0.4, 1.3)	0.6 (-0.3, 1.5)
≥ 4.5	926	87.7 (9.7)	0 (Ref.)	0 (Ref.)
per 0.5 lower			**1.2 (0.6, 1.8)**	**0.8 (0.2, 1.4)**
p-value			**< 0.0001**	**0.01**
**Chinese-American**	679	91.5 (9.9)		
< 4.0	160	90.9 (10.1)	-0.7 (-3.4, 1.9)	-1.6 (-6.0, 2.7)
4.0–4.49	435	91.5 (9.7)	-0.8 (-3.2, 1.6)	-1.7 (-5.5, 2.1)
≥ 4.5	84	92.5 (10.7)	0 (Ref.)	0 (Ref.)
per 0.5 lower			0.1 (-1.3, 1.5)	-0.7 (-2.9, 1.5)
p-value			0.92	0.52
**African-American**	1345	90.2 (10.8)		
< 4.0	270	91.3 (11.4)	**1.7 (0.0, 3.3)**	0.6 (-1.7, 2.8)
4.0–4.49	707	89.7 (10.7)	0.0 (-1.3, 1.3)	-1.0 (-2.8, 0.7)
≥ 4.5	368	90.5 (10.4)	0 (Ref.)	0 (Ref.)
per 0.5 lower			**1.2 (0.5, 1.9)**	0.3 (-0.7, 1.4)
p-value			**0.002**	0.56
**Hispanic**	1110	91.0 (10.9)		
< 4.0	136	92.2 (12.5)	2.0 (-0.3, 4.2)	1.7 (-1.2, 4.5)
4.0–4.49	585	90.9 (10.8)	0.9 (-0.4, 2.2)	0.9 (-0.9, 2.6)
≥ 4.5	389	90.8 (10.3)	0 (Ref.)	0 (Ref.)
per 0.5 lower			**1.1 (0.2, 2.1)**	0.6 (-0.6, 1.7)
p-value			**0.02**	0.35

Model 1: adjusted for age (continuous), sex, race, and study site.

Model 2: M1 + waist circumference (continuous), BMI (continuous), smoking, family history of diabetes, education, income, alcohol use, physical activity, systolic blood pressure, antihypertensive medication use and use of other medications listed in Table 1.

Significant associations in bold.

Race*serum potassium, *P* = 0.53

For all 4 measures of K, in minimally-adjusted models (model 1), we found significant inverse associations between K and HOMA-IR, with lower levels of K associated with higher HOMA-IR. However, none of these associations remained significant in fully-adjusted models.(data not shown)

### Incident diabetes

In longitudinal analyses, of the 5415 participants who were free from diabetes at baseline, 1281 participants developed diabetes during a mean follow-up of 8 years. During this time, there was a higher crude incident rate of diabetes (cases per 1000 person-years) among Hispanics (41.6) compared to Chinese Americans (34.0), African Americans (29.2), and whites (26.0).

Serum K was inversely associated with diabetes risk, but this association was attenuated in fully-adjusted models.(Table 3) Compared to those with a higher serum K (≥4.5mmol/L), after adjustment for age, sex, race, and study site, for those participants with a low-normal serum K (<4.0mmol/L), there was a significantly increased risk of diabetes, with an HR (95% CI) of 1.23 (1.04, 1.47), *P*-value = 0.02; however this association was no longer significant with further adjustment. There was no statistical interaction between serum K and ethnic group on this association (*P* = 0.25, model 2). There was also no statistical interaction between serum K and hypertension status In this association (*P* = 0.53) This pattern of association was similar for all ethnic groups except for Chinese Americans.

**Table 3:** Associations Between Serum Potassium and Incident Diabetes, by Ethnicity (N = 5415).

				Hazard ratio (95% CI)	
Serum K (mmol/L)	Number at risk	Number of events	Unadjusted incidence ratio (per 1000 pys)	Model 1	Model 2
**All participants**	5415	1281	30.8		
< 4.0	1767	208	35.0	**1.23 (1.04, 1.47)**	0.96 (0.75, 1.24)
4.0–4.49	804	662	29.8	0.99 (0.87, 1.12)	1.05 (0.89, 1.23)
≥ 4.5	2844	411	30.5	1.0 (Ref.)	1.0 (Ref.)
per 0.5 lower				**1.11 (1.02, 1.20)**	1.01 (0.91, 1.12)
p-value				**0.02**	0.86
**White**	2281	477	26.0		
< 4.0	238	51	28.6	1.22 (0.89, 1.68)	0.98 (0.66, 1.45)
4.0–4.49	1117	235	25.5	1.02 (0.84, 1.23)	1.13 (0.91, 1.42)
≥ 4.5	926	191	25.9	1.0 (Ref.)	1.0 (Ref.)
per 0.5 lower				1.14 (0.99, 1.30)	1.05 (0.91, 1.22)
p-value				0.06	0.49
**Chinese-American**	679	175	34.0		
< 4.0	160	42	34.4	0.85 (0.51, 1.40)	0.47 (0.21, 1.08)
4.0–4.49	435	107	32.2	0.75 (0.48, 1.18)	**0.50 (0.26, 0.97)**
≥ 4.5	84	26	43.0	1.0 (Ref.)	1.0 (Ref.)
per 0.5 lower				0.96 (0.74, 1.25)	0.85 (0.52, 1.39)
p-value				0.77	0.52
**African-American**	1345	290	29.2		
< 4.0	270	63	31.9	1.17 (0.84, 1.63)	0.77 (0.46, 1.30)
4.0–4.49	707	148	27.9	0.96 (0.72, 1.26)	0.95 (0.64, 1.40)
≥ 4.5	368	79	29.8	1.0 (Ref.)	1.0 (Ref.)
per 0.5 lower				1.05 (0.89, 1.23)	0.88 (0.70, 1.11)
p-value				0.56	0.27
**Hispanic**	1110	339	41.6		
< 4.0	136	52	54.0	**1.51 (1.09, 2.08)**	1.27 (0.81, 1.98)
4.0–4.49	585	172	39.4	1.02 (0.80, 1.29)	1.08 (0.79, 1.49)
≥ 4.5	389	115	40.9	1.0 (Ref.)	1.0 (Ref.)
per 0.5 lower				**1.19 (1.00, 1.41)**	1.08 (0.88, 1.33)
p-value				**0.04**	0.46

Model 1: adjusted for age (continuous), sex, race, and study site.

Model 2: M1 + waist circumference (continuous), BMI (continuous), smoking, family history of diabetes, education, income, alcohol use, physical activity, systolic blood pressure, antihypertensive medication use and use of other medications listed in Table 1.

Significant associations in bold. Race*serum potassium, *P* = 0.25

Dietary K was also inversely associated with diabetes risk, but this association, again, was attenuated in fully-adjusted models. For each standard deviation (SD) decrease in dietary K, after adjustment for age, sex, race, and study site, and total energy intake, there was a significantly increased risk of diabetes, with an HR (95% CI) of 1.11 (1.00, 1.24), *P*-value = 0.04.(Table 4) We found no significant associations between random spot urinary K measures and diabetes risk. However, in our minimally-adjusted model, we did find an inverse association between FEK and diabetes risk.(S3 Appendix Table) Compared to those in the highest quartile of FEK, in models adjusted for age, sex, race, and study site only, those in the lowest quartile (<8%), had a higher risk of diabetes (HR) (95% CI) of 1.19 (1.01, 1.39), *P*-value = 0.03; however, this association was attenuated in model 2. There were no significant interactions between dietary K, urinary K or FEK and ethnic group on these associations for any of the models evaluated. Patterns of association between urinary K or FEK with diabetes risk were similar for all 4 ethnic groups.

**Table 4:** Associations Between Dietary Potassium (mg/day) and Incident Diabetes, by Ethnicity (N = 5415).

				Hazard ratio (95% CI)	
Dietary K (mg/Day)	Number at risk	Number of events	Unadjusted incidence ratio (per 1000 pys)	Model 1	Model 2
**All**	5415	1281	30.8		
<1820	1384	309	31.7	1.21 (0.96, 1.53)	0.93 (0.68, 1.27)
1820–2600	1322	305	29.2	1.05 (0.86, 1.28)	0.95 (0.74, 1.22)
2600–3550	1335	325	30.2	1.04 (0.88, 1.24)	0.92 (0.74, 1.14)
>3550	1374	342	32.1	1.0 (Ref.)	1.0 (Ref.)
per SD decrease				**1.11 (1.00, 1.24)**	0.99 (0.86, 1.13)
p-value				**0.04**	0.86
**White**	2281	477	26		
<1820	356	68	24.8	1.28 (0.92, 1.79)	1.17 (0.79, 1.75)
1820–2600	583	115	24.4	1.11 (0.85, 1.45)	1.04 (0.76, 1.43)
2600–3550	690	154	27.6	1.19 (0.93, 1.51)	1.12 (0.85, 1.48)
>3550	652	140	26.2	1.0 (Ref.)	1.0 (Ref.)
per SD decrease				**1.18 (1.04, 1.34)**	1.08 (0.91, 1.27)
p-value				**0.009**	0.38
**Chinese-American**	679	175	34		
<1820	328	84	34.9	1.19 (0.72, 1.96)	0.88 (0.34, 2.25)
1820–2600	147	35	31	0.98 (0.56, 1.69)	0.98 (0.36, 2.61)
2600–3550	128	35	34.6	1.02 (0.59, 1.76)	0.88 (0.33, 2.37)
>3550	76	21	34.9	1.0 (Ref.)	1.0 (Ref.)
per SD decrease				1.18 (0.96, 1.45)	1.20 (0.82, 1.77)
p-value				0.11	0.36
**African-American**	1345	290	29.2		
<1820	401	90	31.8	1.38 (0.94, 2.01)	0.75 (0.43, 1.30)
1820–2600	316	69	28.8	1.20 (0.83, 1.74)	1.01 (0.62, 1.65)
2600–3550	307	62	26.5	0.99 (0.69, 1.43)	0.84 (0.52, 1.37)
>3550	321	69	29.3	1.0 (Ref.)	1.0 (Ref.)
per SD decrease				1.15 (0.99, 1.34)	0.98 (0.81, 1.17)
p-value				0.07	0.79
**Hispanic**	1110	339	41.6		
<1820	237	67	37.9	0.99 (0.70, 1.39)	0.74 (0.47, 1.16)
1820–2600	289	86	39.3	0.92 (0.67, 1.25)	0.71 (0.47, 1.06)
2600–3550	249	74	40.1	0.89 (0.65, 1.21)	0.61 (0.40, 0.93)
>3550	335	112	47.7	1.0 (Ref.)	1.0 (Ref.)
per SD decrease				0.99 (0.87, 1.14)	0.87 (0.74, 1.01)
p-value				0.93	0.07

Model 1: adjusted for age (continuous), sex, race, study site, and total energy intake.

Model 2: M1 + waist circumference (continuous), BMI (continuous), smoking, family history of diabetes, education, income, alcohol use, physical activity, systolic blood pressure, antihypertensive medication use and use of other medications listed in Table 1.

Significant associations in bold.

Race*dietary potassium, *P* = 0.46

## Discussion

In these analyses of a multi-ethnic US cohort, we found a significant inverse association between serum K with fasting glucose. We also found crude inverse associations between K measures, insulin resistance, and diabetes risk; however, these associations were not significant with multivariable-adjustment of confounders. We found no statistical interaction between measures of K and ethnic group on the outcomes of either fasting glucose or diabetes risk. Some of the effect estimates of these associations, particularly involving serum K, appeared to differ by ethnicity in direction and/or magnitude, but these differences were not statistically significant. These differences were most notable among the Chinese Americans, a population in whom these associations have not been well-studied before.

The finding of a significant inverse association between serum K and fasting glucose is consistent with other cross-sectional studies which evaluated associations between K and measures of glucose metabolism. In analyses of the Cardiovascular Health Study (CHS), compared to those with higher levels, those with lower serum and dietary K had reduced insulin sensitivity; this study did not evaluate the association between K and fasting glucose.[12] A cross-sectional study of a German cohort found that participants with a low-normal serum K had a higher prevalence of prediabetes among the participants with hypertension.[13]

The finding of no significant associations between measures of K and diabetes risk differs from some studies which did show a significant inverse association between serum or dietary K and diabetes risk.[1–3] In the analyses of the Atherosclerotic Risk in Communities (ARIC) and Coronary Artery Risk Development in Young Adults (CARDIA) cohorts, significant associations were found between serum K and dietary K intake with diabetes risk, respectively.[1, 2] In the ARIC cohort, compared with those with a high-normal serum potassium level (5.0–5.5mmol/L), adults with serum K <4.0mmol/L, 4.0 -< 4.5mmol/L, and 4.5 -< than 5.0mmol/L had an adjusted HR (95% confidence interval [CI]) of incident DM of 1.64 (95% CI, 1.29–2.08), 1.64 (95% CI, 1.34–2.01), and 1.39 (95% CI, 1.14–1.71), respectively.[1] In the CARDIA cohort, there was a statistical interaction between dietary K intake and race (black/white). The association between dietary K intake and diabetes risk was not significant for whites; however, the association between dietary K intake, based in diet histories, and diabetes risk was non-linear but significant for blacks. In the CARDIA cohort, compared with black participants in the highest quintile of dietary K intake, and with adjustment for dietary factors and diabetes risk factors, those in the lowest to higher dietary K intake quintile had adjusted HRs (95% CI) of incident diabetes of 1.72 (0.87, 3.42), 1.99 (1.04, 3.78), 2.10 (1.14, 3.87), and 1.46 (0.79, 2.68) respectively.[2] In a sub-cohort of CARDIA, a significant inverse association was also found between 24-hour urinary measures and diabetes risk.[2] Compared to both ARIC and CARDIA cohorts, the participants of the MESA cohort were, on average, older in age. The MESA cohort has the strength of having a diverse population, but the number of participants in each specific minority ethnic group is smaller than the number of African Americans in either ARIC or CARDIA.[1, 2] In the analyses of the ARIC and CARDIA cohorts, respectively, over 2700 and 2300 African Americans were included in the analyses, while in these analyses of the MESA cohort, there were half this number of African Americans and even fewer Hispanics and Chinese Americans. The smaller population sizes could have limited the detection of a significant association between K measures and diabetes risk in the higher risk populations of MESA. Further study is needed to determine the potential differences in these cohorts that could account for these differing results.

This study extends prior studies by examining associations of K with glucose and diabetes among Hispanics and Chinese Americans. Among Hispanics, inverse associations between serum K with fasting glucose and diabetes risk were similar to those among white and black MESA participants. In contrast, among Chinese Americans, these associations were reversed; however, these associations were also not significant and sample size was small. These potential variations in associations by ethnic group deserve further study.

In multivariate models, urinary K was not significantly associated with fasting glucose or incident diabetes. However, in minimally-adjusted models, there was an inverse association between urine K with HOMA-IR. Random spot urine specimens are easy to collect during study visits, but their use for study measures likely needs further study for validation. 24-hour urinary potassium measurements have been found to be a fairly accurate reflection of dietary potassium intake;[14] however, 24-hour urine samples are very difficult to collect and have their own measurement problems.[15, 16] In the CARDIA study, three consecutive 24-hour urine specimens were collected in a sub-cohort of the participants. In this sub-cohort, those in the lowest quintile of 24-hour urinary K had a significantly increased diabetes risk compared to those participants in the highest quintile of 24-hour urinary K (HR 2.45; 95% CI 1.08, 5.59; p for trend = 0.04).[2] Recently in an international cohort, 24-hour urinary K levels were estimated from morning fasting midstream urine samples.[17, 18] In this study, investigators found a significant association between lower urinary K levels and increased systolic blood pressure an increase in a composite outcome of all-cause mortality and major cardiovascular events. This study did not evaluate the associations of urinary K with diabetes risk or measures of glucose metabolism. In our analyses of MESA participants, random samples were used for measurement of urinary K. Given the diurnal variation in urinary K excretion, the use of spot urine samples may need to be standardized to yield the best estimate of 24-hour urinary K measures.[19, 20]

In theory, FEK should reflect the renal handling of filtered potassium, as influenced by aldosterone as well as dietary K and sodium intake as well as serum K. In minimally-adjusted models only, our analyses did reveal inverse associations between FEK with fasting glucose, HOMA-IR, and diabetes risk, which may reflect these influences. In future studies, it may be important to assess the individual effect of these influences, such as measures of aldosterone, directly on measures of glucose, insulin, and diabetes risk, to help determine if and how FEK can be used in future studies.

There are several limitations of these analyses that should be mentioned. Limitations of this study include the measurement of serum and urine K at a single time point. Serum K and indeed all biological analytes have inherent variability that can lead to misclassification of exposures. While serum K is tightly regulated with homeostatic mechanisms within a fairly tight range, there is within-person variation that is significant with within-person coefficients of variation of about 5%.[21, 22] While this within-person variability is reasonably low, the within-population range is relatively small, and so persons could easily fluctuate between categories. Another limitation of this study is in the definition of the diabetes outcome which is limited by the dataset to use of fasting glucose and use of medications; post-challenge glucose and hemoglobin A1c measures were not available. Compared to the participants included in our analyses, the baseline characteristics of the participants who were excluded from our analyses differed somewhat with regards to demographics. We found no significant differences in K measures or fasting glucose. Other differences were modest and would be unlikely to bias the conclusions drawn from our analyses.

Given that this is a prospective cohort study, a post-hoc power calculation is generally not appropriate. However, given the relatively large number of events of incident diabetes and the large population, while modest associations, particularly within each ethnic sub-group, could be missed, it is unlikely that large associations would be missed. While there were no significant associations found between measures of K and diabetes risk in this cohort, there have been significant associations found in other cohorts. There could be other factors that mediate associations between K and glucose metabolism that we were unable to determine from this dataset, including the presence of genetic susceptibility. Further study may be warranted to take into account genetic susceptibility particularly with regards to the presence of mutations within genes of K-channels such as KCNJ11.

## Conclusions

In conclusion, these analyses of the MESA cohort identified significant although small inverse associations between serum K and fasting glucose. There was no significant association between K and diabetes risk. While we found no statistical interaction between K and ethnicity, we did find differences in these associations that varied in magnitude and direction by ethnic group. Given the potential importance of K measures on not only diabetes risk, but also cardiovascular health, further study is needed to describe, more precisely, the relationship of potassium with glucose metabolism and potential effects on longer-term health outcomes. Also further study is needed to determine if these associations are similar in different ethnic groups.

## Supporting Information

S1 Appendix Table Associations Between Dietary Potassium and Fasting Glucose, by Ethnicity (N = 5415). (DOCX)

S2 Appendix Table Associations Between Fractional Excretion (%) (FEK) of Potassium and Fasting Glucose, by Ethnicity. (DOCX)

S3 Appendix Table Associations Between Fractional Excretion (%) of Potassium (FEK) and Incident Diabetes, by Ethnicity. (DOCX)

S4 Appendix Table Baseline Characteristics of non-diabetic MESA participants included and excluded from analysis population. (DOCX)

## Abbreviations

ARIC: Atherosclerotic Risk in Communities study
BMI: body mass index
CARDIA: Coronary Artery Risk Development in Young Adults
FEK: fractional excretion of potassium
FFQ: food frequency questionnaire
HOMA-IR: homeostasis model assessment of insulin resistance
IRAS: Insulin Resistance and Atherosclerosis Study
K: Potassium
MESA: Multi-Ethnic Study of Atherosclerosis Study
