# Supplementary material for: Potassium Measures and Their Associations with Glucose and Diabetes Risk: The Multi-Ethnic Study of Atherosclerosis (MESA)
Source: PLoS One. 2016 Jun 9;11(6):e0157252. doi: 10.1371/journal.pone.0157252 (PMC4900670; doi:10.1371/journal.pone.0157252)
Supplement: S1 Appendix Table — (DOCX) [file pone.0157252.s001.docx]

**S1 Appendix Table. Associations Between Dietary Potassium and Fasting Glucose, by Ethnicity (N = 5415).**

|  |  |  | **Adjusted difference (95% CI) (mg/dL)** | |
| --- | --- | --- | --- | --- |
| **Dietary K (mg/day)** | **N** | **Mean glucose (mg/dL) (SD)** | **Model 1** | **Model 2** |
| **All** | 5415 | 89.5 (10.5) |  |  |
| <1820 | 1384 | 89.8 (10.3) | **1.5 (0.3, 2.6)** | 0.1 (-1.3, 1.4) |
| 1820-2600 | 1322 | 89.4 (11.0) | **1.1 (0.1, 2.0)** | -0.3 (-1.5, 0.8) |
| 2600-3550 | 1335 | 89.4 (10.3) | 0.7 (-0.2, 1.5) | -0.2 (-1.2, 0.8) |
| >3550 | 1374 | 89.6 (10.5) | 0 (Ref.) | 0 (Ref.) |
| per SD decrease |  |  | **1.1 (0.6, 1.6)** | 0.5 (-0.1, 1.1) |
| p-value |  |  | **< 0.0001** | 0.10 |
| **White** | 2281 | 87.8 (10.1) |  |  |
| <1820 | 356 | 86.9 (9.7) | 1.1 (-0.3, 2.5) | 0.3 (-1.3, 1.9) |
| 1820-2600 | 583 | 87.3 (10.6) | 0.6 (-0.6, 1.8) | -0.4 (-1.7, 0.9) |
| 2600-3550 | 690 | 88.4 (10.1) | 1.2 (0.1, 2.3) | 0.5 (-0.7, 1.7) |
| >3550 | 652 | 88.1 (9.9) | 0 (Ref.) | 0 (Ref.) |
| per SD decrease |  |  | **1.1 (0.5, 1.6)** | 0.6 (-0.1, 1.3) |
| p-value |  |  | **0.0003** | 0.08 |
| **Chinese-American** | 679 | 91.5 (9.9) |  |  |
| <1820 | 328 | 91.3 (9.6) | **2.6 (0, 5.1)** | -1.2 (-5.9, 3.6) |
| 1820-2600 | 147 | 91.9 (10.5) | 2.4 (-0.4, 5.2) | 1.2 (-3.8, 6.1) |
| 2600-3550 | 128 | 92.2 (10.0) | 2.6 (-0.2, 5.4) | 0.0 (-4.9, 4.9) |
| >3550 | 76 | 90.2 (10.3) | 0 (Ref.) | 0 (Ref.) |
| per SD decrease |  |  | **1.5 (0.5, 2.4)** | 0.1 (-1.5, 1.7) |
| p-value |  |  | 0.002 | 0.86 |
| **African-American** | 1345 | 90.2 (10.8) |  |  |
| <1820 | 401 | 90.7 (10.9) | **2.9 (1.0, 4.7)** | 1.1 (-1.3, 3.5) |
| 1820-2600 | 316 | 90.2 (11.1) | 2.1 (0.3, 3.9) | -0.1 (-2.4, 2.3) |
| 2600-3550 | 307 | 89.8 (10.5) | 0.7 (-1.0, 2.4) | -0.3 (-2.5, 1.9) |
| >3550 | 321 | 90.1 (10.5) | (0) Ref. | 0 (Ref.) |
| per SD decrease |  |  | **1.7 (1.0, 2.4)** | **1.0 (0.1, 1.8)** |
| p-value |  |  | **< 0.0001** | **0.02** |
| **Hispanic** | 1110 | 91.0 (10.9) |  |  |
| <1820 | 237 | 90.3 (10.5) | 0.1 (-1.8, 2.1) | -1.1 (-3.8, 1.5) |
| 1820-2600 | 289 | 91.5 (11.3) | 0.7 (-1.2, 2.5) | -0.9 (-3.3, 1.5) |
| 2600-3550 | 249 | 90.0 (10.3) | -1.2 (-2.9, 0.6) | **-2.7 (-4.9, -0.5)** |
| >3550 | 335 | 92.0 (11.2) | 0 (Ref.) | 0 (Ref.) |
| per SD decrease |  |  | 0.5 (-0.2, 1.3) | 0.0 (-1.0, 1.0) |
| p-value |  |  | 0.19 | 0.99 |

Model 1: adjusted for age (continuous), sex, race, study site, and total energy intake.

Model 2: M1 + waist circumference (continuous), BMI (continuous), smoking, family history of diabetes, education, income, alcohol use, physical activity, systolic blood pressure, antihypertensive medication use and use of other medications listed in Table 1.

Significant associations in bold.

Race*dietary potassium, *P* =0.18
