# Supplementary material for: Potassium Measures and Their Associations with Glucose and Diabetes Risk: The Multi-Ethnic Study of Atherosclerosis (MESA)
Source: PLoS One. 2016 Jun 9;11(6):e0157252. doi: 10.1371/journal.pone.0157252 (PMC4900670; doi:10.1371/journal.pone.0157252)
Supplement: S2 Appendix Table — (DOCX) [file pone.0157252.s002.docx]

**S2 Appendix Table. Associations Between Fractional Excretion (%) (FEK) of Potassium and Fasting Glucose, by Ethnicity.**

|  |  |  | **Adjusted difference (95% CI) (mg/dL)** | |
| --- | --- | --- | --- | --- |
| **FEK (%)** | **N** | **Mean glucose (mg/dL) (SD)** | **Model 1** | **Model 2** |
| **All participants** | 5415 | 89.5 (10.5) |  |  |
| < 8 | 1492 | 89.6 (11.0) | 0.7 (-0.1, 1.5) | 0.3 (-0.7, 1.2) |
| 8 – 11 | 1340 | 89.5 (10.3) | 0.0 (-0.7, 0.8) | -0.1 (-1.0, 0.8) |
| 11 – 14 | 1478 | 89.7 (10.4) | 0.1 (-0.7, 0.9) | -0.4 (-1.3, 0.5) |
| > 14 | 1098 | 89.3 (10.4) | 0 (Ref.) | 0 (Ref.) |
| per 5 unit decrease |  |  | **0.3 (0.0, 0.5)** | 0.0 (-0.4, 0.3) |
| p-value |  |  | **0.08** | 0.97 |
| **White** | 2281 | 87.8 (10.1) |  |  |
| < 8 | 440 | 87.6 (10.3) | 1.0 (-0.1, 2.2) | -0.2 (-1.5, 1.1) |
| 8 – 11 | 557 | 88.5 (10.6) | 1.1 (0.1, 2.2) | 0.5 (-0.7, 1.7) |
| 11 – 14 | 490 | 88.2 (9.8) | 0.7 (-0.3, 1.8) | 0.1 (-1.0, 1.3) |
| > 14 | 790 | 87.2 (9.9) | 0 (Ref.) | 0 (Ref.) |
| per 5 unit decrease |  |  | 0.4 (0.0, 0.7) | -0.1 (-0.5, 0.3) |
| p-value |  |  | 0.07 | 0.56 |
| **Chinese-American** | 679 | 91.5 (9.9) |  |  |
| < 8 | 127 | 90.3 (9.9) | -1.1 (-3.3, 1.1) | -1.8 (-5.4, 1.8) |
| 8 – 11 | 181 | 91.4 (9.8) | -0.4 (-2.4, 1.5) | 0.3 (-3.0, 3.6) |
| 11 – 14 | 149 | 91.0 (9.2) | -1.4 (-3.4, 0.5) | -2.8 (-5.9, 0.2) |
| > 14 | 221 | 92.5 (10.5) | 0 (Ref.) | 0 (Ref.) |
| per 5 unit decrease |  |  | -0.2 (-0.9, 0.6) | -0.5 (-1.9, 0.9) |
| p-value |  |  | 0.66 | 0.46 |
| **African-American** | 1345 | 90.2 (10.8) |  |  |
| < 8 | 485 | 90.5 (11.1) | 1.5 (-0.1, 3.1) | 1.0 (-1.4, 3.3) |
| 8 – 11 | 399 | 89.9 (10.2) | 0.3 (-1.4, 1.9) | -0.6 (-3.0, 1.7) |
| 11 – 14 | 236 | 90.6 (11.5) | 0.7 (-1.2, 2.7) | -0.8 (-3.4, 1.8) |
| > 14 | 224 | 90.0 (10.2) | 0 (Ref.) | 0 (Ref.) |
| per 5 unit decrease |  |  | **0.8 (0.1, 1.4)** | 0.5 (-0.4, 1.4) |
| p-value |  |  | 0.02 | 0.24 |
| **Hispanic** | 1110 | 91.0 (10.9) |  |  |
| < 8 | 288 | 91.0 (11.9) | -0.4 (-2.2, 1.5) | 0.3 (-2.2, 2.7) |
| 8 – 11 | 341 | 89.8 (10.2) | -2.2 (-3.8, -0.5) | -1.4 (-3.6, 0.7) |
| 11 – 14 | 223 | 91.2 (11.0) | -1.1 (-2.9, 0.7) | -0.8 (-3.3, 1.7) |
| > 14 | 257 | 92.6 (10.3) | 0 (Ref.) | 0 (Ref.) |
| per 5 unit decrease |  |  | -0.3 (-1.1, 0.4) | 0.1 (-0.9, 1.1) |
| p-value |  |  | 0.37 | 0.79 |

Model 1: adjusted for age (continuous), sex, race, study site.

Model 2: M1 + waist circumference (continuous), BMI (continuous), smoking, family history of diabetes, education, income, alcohol use, physical activity, systolic blood pressure, antihypertensive medication use and use of other medications listed in Table 1.

Significant associations in bold.

Race*FEK, *P* =0.19
