# Supplementary material for: Potassium Measures and Their Associations with Glucose and Diabetes Risk: The Multi-Ethnic Study of Atherosclerosis (MESA)
Source: PLoS One. 2016 Jun 9;11(6):e0157252. doi: 10.1371/journal.pone.0157252 (PMC4900670; doi:10.1371/journal.pone.0157252)
Supplement: S4 Appendix Table — (DOCX) [file pone.0157252.s004.docx]

**S4. Appendix Table. Baseline Characteristics of non-diabetic MESA participants included and excluded from analysis population.**

| **Covariate** | **Excluded from analysis population because of missing K**  **(N = 540)** | **Included in analysis population**  **(N = 5415)** |
| --- | --- | --- |
| **Demographics** |  |  |
| Age (years) | 61.1 ± 10.3 | 61.8 ± 10.3 |
| Sex |  |  |
| Female | 267 (49) | 2924 (54) |
| Male | 273 (51) | 2491 (46) |
| Race/Ethnicity |  |  |
| White, Caucasian | 183 (34) | 2281 (42) |
| Chinese-American | 19 (4) | 679 (13) |
| Black, African-American | 216 (40) | 1345 (25) |
| Hispanic | 122 (23) | 1110 (20) |
| Site |  |  |
| Wake Forest | 122 (23) | 828 ( 15) |
| Columbia | 123 (23) | 827 (15) |
| Johns Hopkins | 139 (26) | 807 (15) |
| Minnesota | 73 (14) | 869 (16) |
| Northwestern | 20 (4) | 1044 (19) |
| UCLA | 63 (12) | 1040 (19) |
| **Anthropometric measures:** |  |  |
| Body mass index (kg/m^2^) | 29.4 ± 6.1 | 27.9 ± 5.3 |
| Waist circumference (cm) | 100.7 ± 15.3 | 96.8 ± 14.0 |
| **Lifestyle and family history:** |  |  |
| Smoking status |  |  |
| Never | 236 (44) | 2762 (51) |
| Former | 195 (36) | 1967 (36) |
| Current | 96 (18) | 679 (13) |
| Family history of DM | 221 (41) | 2086 (39) |
| Education |  |  |
| Less than high school degree | 88 (16) | 881 (16) |
| High school to some college | 225 (42) | 1821 (34) |
| College degree or higher | 214 (40) | 2705 (50) |
| Total gross family income, $ |  |  |
| < 20,000 | 189 (35) | 1850 (34) |
| 20,000 – 49,999 | 209 (39) | 2099 (39) |
| ≥ 50,000 | 94 (17) | 1290 (24) |
| Number of alcoholic drinks/week |  |  |
| 0 | 160 (30) | 1313 (24) |
| 1-7 | 130 (24) | 1539 (28) |
| > 7 | 42 (8) | 549 (10) |
| Physical activity |  |  |
| High activity | 143 (26) | 1312 (24) |
| Moderate activity | 264 (49) | 2796 (52) |
| Light activity | 121 (22) | 1302 (24) |
| **Medical history:** |  |  |
| Systolic blood pressure (mmHg) | 128.0 ± 22.0 | 125.4 ± 21.2 |
| Diastolic blood pressure (mmHg) | 73.2 ± 10.3 | 71.8 ± 10.2 |
| Fasting serum glucose (mg/dL) | 90.3 ± 10.9 | 89.5 ± 10.5 |
| Antihypertensive medication use | 198 (37) | 1789 (33) |
| ARB or ACE-I use | 98 (18) | 749 (14) |
| Thiazide diuretics w/o K-sparing agents | 40 (7) | 351 (6) |
| Thiazide diuretics w/ K-sparing agents | 25 (5) | 203 (4) |
| Loop diuretics | 8 (1) | 79 (1) |
| Potassium supplements | 9 (2) | 97 (2) |
| Potassium-sparing agents | 3 (1) | 28 (1) |
| eGFR CKD-EPI (mL/min/1.73m^2^) | 77.2 ± 17.1 | 77.8 ± 15.5 |
| Dietary potassium intake (mg/day) | 2754 ± 1544 | 2847 ± 1458 |

Entries are mean ± standard deviation or number (%)

ARB- angiotensin II receptor antagonists; ACE-I- angiotension converting enzyme-inhibitors

GFR= glomerular filtration rate
